# Coverage of intermittent preventive treatment of malaria in infants after four years of implementation in Sierra Leone

**DOI:** 10.1186/s12936-023-04575-6

**Published:** 2023-05-02

**Authors:** Augustin E. Fombah, Haily Chen, Kwabena Owusu-Kyei, Llorenç Quinto, Raquel Gonzalez, Julian Williams, Mireia LLach Berne, Myrte Wassenaar, Abubakarr Jalloh, Joe-Henry C. Sunders, Maximo Ramirez, Cesc Bertran-Cobo, Francisco Saute, Didier K. Ekouevi, Valérie Briand, Anitta R. Y. Kamara, Tom Sesay, Mohamed Samai, Clara Menendez

**Affiliations:** 1 Barcelona Institute for Global Health, Hospital Clinic-University of Barcelona, Barcelona, Spain; 2 Ministry of Health and Sanitation, Freetown, Sierra Leone; 3 University Medical Center Utrecht - Utrecht University, Utrecht, The Netherlands; 4 Manhiça Health Research Center, Manhiça, Mozambique; 5 College of Medicine and Allied Health Sciences, University of Sierra Leone, Freetown, Sierra Leone; 6 Université de Lomé, Lomé, Togo; 7 National Institute for Health and Medical Research (INSERM) UMR 1219, Research Institute for Sustainable Development (IRD) EMR 271, Bordeaux Population Health Centre, University of Bordeaux, Bordeaux, France; 8 National Malaria Control Program, Directorate of Disease Prevention and Control, Ministry of Health and Sanitation, Freetown, Sierra Leone; 9 Directorate of Research and Training, Ministry of Health and Sanitation, Freetown, Sierra Leone; 10 Ministry of Health and Sanitation, Directorate Research and Training, Freetown, Sierra Leone

**Keywords:** Malaria prevention, Child health, IPTi, PMC, Sub-Saharan Africa, Sierra Leone

## Abstract

**Background:**

Intermittent Preventive Treatment of malaria in infants (IPTi) is a malaria control strategy consisting of the administration of an anti-malarial drug alongside routine immunizations. So far, this is being implemented nationwide in Sierra Leone only. IPTi has been renamed as Perennial Malaria Chemoprevention -PMC-, accounting for its recently recommended expansion into the second year of life. Before starting a pilot implementation on PMC, the currently implemented strategy and malaria prevalence were assessed in young children in selected areas of Sierra Leone.

**Methods:**

A cross-sectional, community-based, multi-stage cluster household survey was conducted from November to December 2021 in selected districts of the Northern and northwestern provinces of Sierra Leone among 10–23 months old children, whose caretakers gave written informed consent to participate in the survey. Coverage of IPTi and malaria prevalence—assessed with rapid diagnostic tests—were calculated using percentages and 95% confidence intervals weighted for the sampling design and adjusted for non-response within clusters. Factors associated with RDT + and iPTi coverage were also assessed.

**Results:**

A total of 720 children were recruited. Coverage of three IPTi doses was 50.57% (368/707; 95% CI 45.38–55.75), while prevalence of malaria infection was 28.19% (95% CI 24.81–31.84). Most children had received IPTi1 (80.26%, 574/707; 95% CI 75.30–84.44), and IPTi2 (80.09%, 577/707; 95% CI 76.30–83.40) and over half of the children also received IPTi3 (57.72%, 420/707; 95% CI 53.20–62.11). The uptake of each IPTi dose was lower than that of the vaccines administered at the same timepoint at all contacts.

**Conclusion:**

In Sierra Leone, half of the children received the three recommended doses of IPTi indicating an increase in its uptake compared to previous data of just a third of children receiving the intervention. However, efforts need to be made in improving IPTi coverage, especially in the planned expansion of the strategy into the second year of life following recent WHO guidelines.

**Supplementary Information:**

The online version contains supplementary material available at 10.1186/s12936-023-04575-6.

## Background

Despite global efforts for malaria control, the infection continues to have a devastating impact on people’s health in endemic areas with a global estimate of 247 million malaria cases and 619,000 malaria deaths in 2021 compared to 245 million malaria cases and 625,000 malaria deaths in 2020 [1]. Sub-Saharan Africa (SSA) continues to carry the heaviest malaria burden, accounting in 2021 for about 95% of malaria cases [1]. While 96% of global malaria deaths occurred in 29 countries, four sub-Saharan African countries accounted for over half of the global deaths reported. Between 2019 and 2021, there were 63,000 deaths that were due to disruptions to essential malaria services during the COVID-19 pandemic [1]. Nearly 80% of malaria deaths from 2015 to 2021 occurred in children less than 5 years of age (U5).

In 2010, the World Health Organization (WHO) recommended Intermittent Preventive Treatment for malaria in infants (IPTi) to control the infection in infants living in areas with moderate-to-high malaria transmission and with low parasitic resistance to sulfadoxine-pyrimethamine (SP) (2). IPTi consists of the administration of a full therapeutic course of SP at 10 and 14 weeks of age, and 9 months of age alongside routine immunizations through the Expanded Programme on Immunization (EPI) [2].

Despite IPTi has been shown to be safe and efficacious in reducing clinical malaria, anaemia, and all-cause hospital admissions, to date Sierra Leone is the only country to have implemented this intervention nationwide since 2018 [3–5] [6]. In 2018, a community-based household survey conducted in Kambia district reported that about a third (32.2%) of eligible infants had received the recommended three doses of IPTi [6]. Thus, even where IPTi is policy, important limitations exist about its coverage and therefore, effectiveness.

As part a of a multi-country pilot project (MULTIPLY- MULTIple doses of IPTi Proposal: a Lifesaving high Yield intervention), to promote and expand IPTi implementation that includes Sierra Leone [7] a household survey (HHS) was conducted to assess IPTi coverage, and malaria prevalence in children under 2 years of age (U2) living in project districts in the country. This information is key for future assessment of the impact of the IPTi and the implications for its planned expansion.

IPTi has been recently renamed by the WHO as Perennial Malaria Chemoprevention (PMC), accounting for its expansion into the second year of life given the added EPI contacts during this period [8]. Mozambique recently announced adoption of the PMC policy and has started a pragmatic implementation in some districts**.**

## Methods

### Survey population and inclusion/exclusion criteria

Children aged 10 to 23 months inclusive at the moment of the interview, whose caretaker agreed to participate in the survey by signing an informed consent and who lived in MULTIPLY project districts of Sierra Leone (Bombali, Port Loko, and Tonkolili) were the targeted population for the HHS. Malaria prevalence in children U5 in 2016 was 38%, 59%, and 56%, in these three districts, respectively [9].

### Sample size

According to the latest available estimate of 32.2% IPTi coverage [6], it was deemed that 710 children were needed to assess coverage rates with 95% confidence and 5% precision, assuming a design effect of 2 and a 10% of non-response. Assuming that for every five households one child would meet the inclusion criteria, it was estimated that a total of 3550 households were needed to be visited to achieve the recruitment goal.

### Sampling methods

**Fig. 1:**
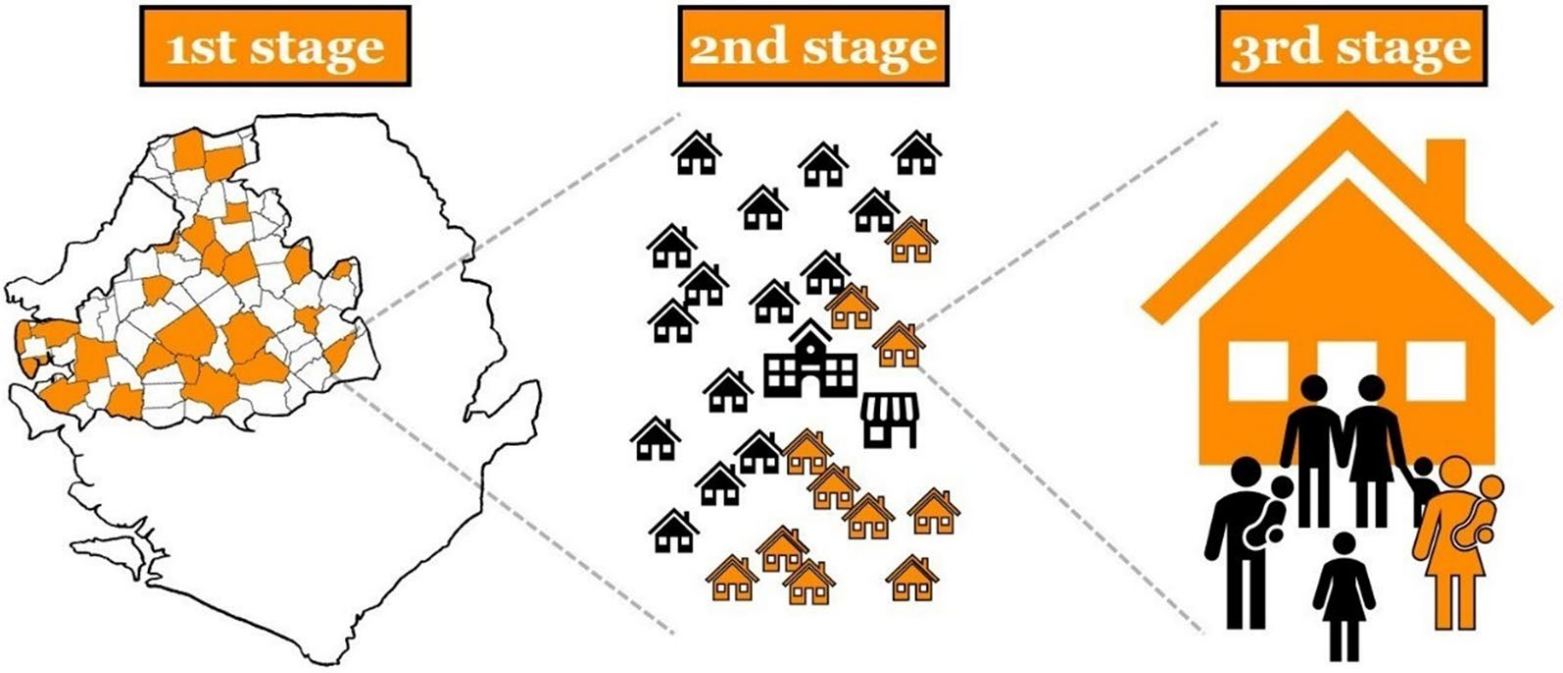
MULTIPLY Baseline Household Survey multi-stage sampling methodology. Legend.  Randomly selected

This was a cross-sectional, community-based, multi-stage cluster HHS. Sample selection was undertaken in three stages as follows (Additional file 1: Annex 1, Fig. 1), firstly, a random selection of clusters within project areas; secondly, a random selection of households within selected clusters; and thirdly, a random selection of children amongst all eligible ones living in each selected and visited household. This approach was adapted from the Malaria Indicator Survey and the EPI sampling method [10, 11]. A more detailed sampling methodology is explained in Additional file 1: Annex 1.

Briefly, the first sampling stage was conducted before survey implementation in collaboration with Statistics Sierra Leone, which provided a list of clusters in project areas based on the 2015 national census. The number of clusters to be selected was determined by dividing the number of children to be included in the survey (N = 710) by the number of children to be interviewed in each cluster. For feasibility reasons, the cluster size was 12 children per day per cluster [11]. Therefore, 60 clusters were selected applying probability proportional to size (PPS) sampling, and recruitment was increased to 720 children to enable an even enrolment of 12 children per cluster. Additionally, six backup clusters were selected in case any of the 60 main clusters were inaccessible to data collection. The second and third sampling stages were done by field teams during data collection (Fig. 1).

### Data collection

Information on IPTi administration was recorded directly from the EPI card except when this was not available in which case it was obtained by asking the child’s caretaker. A malaria rapid diagnostic test (RDT) was performed in all children regardless of symptoms or signs of malaria; if the RDT was positive anti-malarial treatment was provided and children were referred following national guidelines for malaria treatment and referral.

Electronic devices were used for data collection onto the REDCap Mobile Android App [12, 13]. Once a cluster was completed, data on the tablets were reviewed, saved, and sent to the server before starting with another cluster. Paper-based tools were completed to facilitate data validation after data collection. All paper-based and electronic data were double-checked by the team supervisor to identify and correct mistakes. Validated electronic records were then uploaded to the online server.

### Data management and cleaning

Online data quality rules were executed on mobile devices during the interview and data entry process, and a set of data quality rules executed once new data were uploaded in the server. Periodic data quality and progress reports were produced with the last data received. Reports computed survey profiles for each district based on uploaded data were crossed-checked with the paper-based survey profile completed manually to identify inconsistencies or missing records.

### Statistical analysis

Statistical analyses were conducted with weighted data to account for the complex sampling design (see methodology described in Additional file 1: Annex 1). Weighted estimates were calculated by considering the sampling weights with poststratification weights adjusted for nonresponse and under- or overrepresented districts of the target population.

Descriptive statistics were used to summarize baseline characteristics. Categorical variables were reported in frequencies and weighted percentages. Continuous variables were described in weighted mean or median, depending on the distribution of the variable, and weighted standard deviation (SD) or interquartile range (IQR), respectively.

Malaria prevalence and IPTi coverage were determined using weighted percentages and 95% CIs. Malaria prevalence was defined as the proportion of children with a positive malaria RDT result; Prevalence of clinical malaria was defined as the proportion of children with positive malaria RDT result plus fever (axillary temperature ≥ 37.5 °C) or history of fever in the last 24 h.

IPTi coverage was defined under two scenarios: (i) IPTi administered irrespective of the EPI contact with complete IPTi coverage being defined as the proportion of children who received the recommended three doses of IPTi.; and scenario (ii) whereby IPTi is administered at the recommended EPI contacts, i.e. at 10 weeks (IPTi 1), 14 weeks (IPTi 2), and 9 months (IPTi 3) of age, respectively. In this case the number of children who received IPTi3 does not necessarily represent complete coverage. Thus, some children may have received IPTi at nine months, being recorded as IPTi 3 for registration purposes, but actually they had missed the first and/or the second IPTi doses.

Multilevel mixed-effects logistic regression accounting for nesting of participants within clusters and households was used to identify variables associated with a positive RDT result as well as having received the three IPTi doses. Univariable and multivariable-adjusted three-level random-intercept models with households nested within clusters were estimated using variables selected a priori. Poststratification adjustment were not applied to the sampling-weights for the model estimations since multilevel models require separate weights for each stage-level. Therefore, these regression models were estimated by complete-case analysis accounting for the complex multistage sample design. The assumption that data were missing completely at random (MCAR) was assessed using the regression-based approach suggested by Rouzinov S. and Berchtold A [14]. Odds ratios (ORs) and 95% CIs were reported.

All statistical analyses were performed using Stata, version 17 (Stata Corporation, College Station, Texas, 2021) [15]; all statistical tests were 2-sided, and a *p-*value < 0.05 was considered statistically significant.

## Results

### Recruitment 

**Fig. 2:**
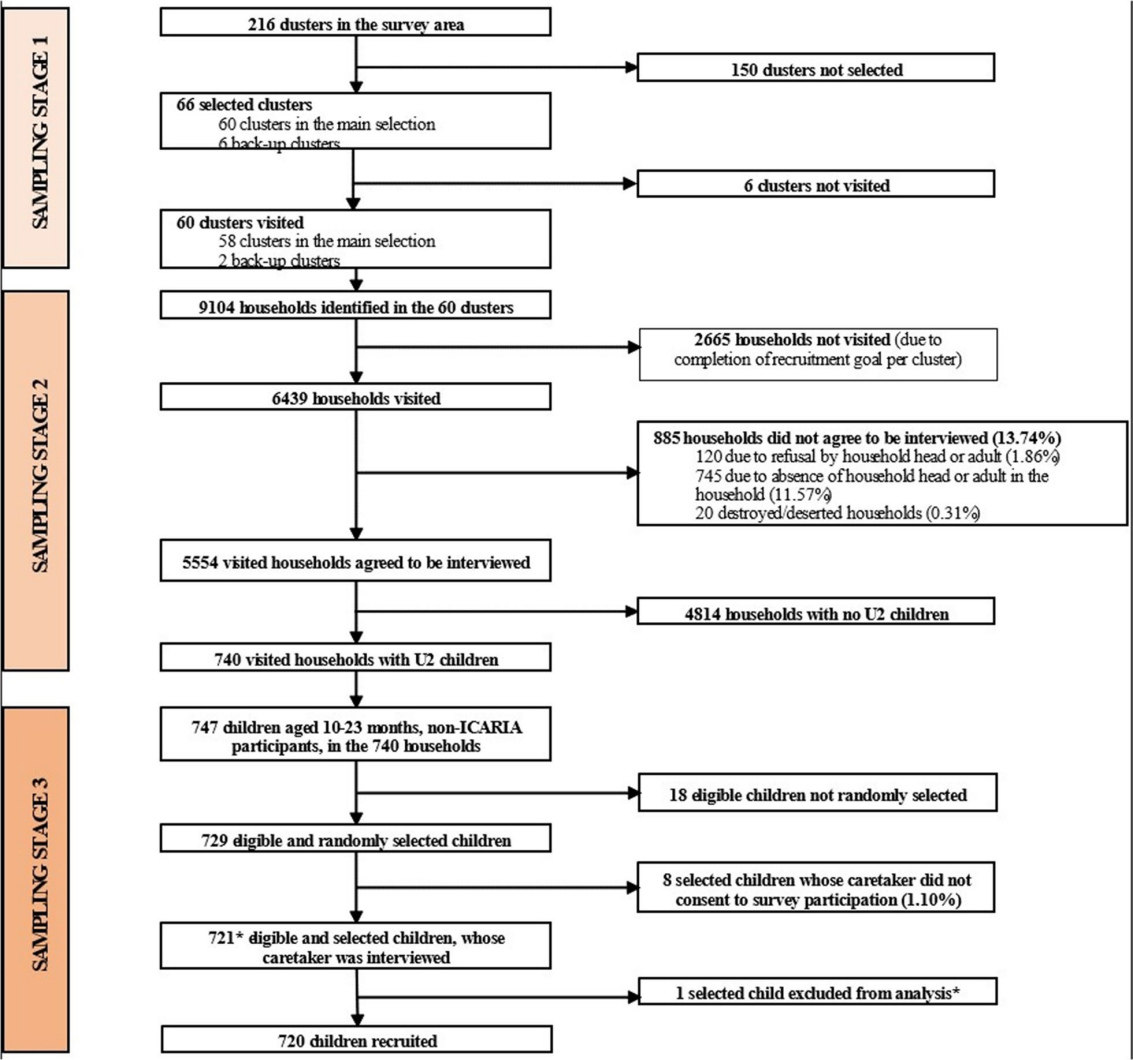
MULTIPLY Baseline Household Survey flowchart. Footnote. *In one cluster, 13 eligible children were recruited instead of the planned 12. Among them, one child was found to have attended EPI in a foreign country, which was not an exclusion criterion at the moment. For ethical consideration, the recruitment was completed and, as a result, an additional child was recruited in the cluster. After careful consideration, this participant was excluded from the statistical analysis, since their inclusion would not comply with the survey’s objectives

From November to December 2021, 9,104 households were listed from 60 randomly selected clusters in project areas (Fig. 2) and of these, 6439 (70.72%) households were visited. In 5554 (86.26%) of the households visited, consent was granted, being U2 children identified in 740 (13.32%) of these households. Caretakers´ interviews were conducted for 721 enrolled children (98.90%) out of 729 eligible ones, while 8 (1.10%) caretakers refused to consent to the interview. One child was found to have attended the EPI clinic in another country and was excluded from the analysis since their inclusion would not comply with the survey’s main objective. Therefore, 720 children were included in the analysis. The non-response rate was 13.74% at the household level.

### Participants characteristics

Baseline characteristics of recruited children, their caretakers, and the household heads are presented in Table 1. Of the 720 children, 50.85% were female. Mean age was 17 months and the average time a child had been residing in the household was 15 months.

**Table 1:** Sociodemographic characteristics of the sample

Enrolled children		n/N	Weighted Percentage or mean ± SD
Child age (in months)			16.68 $\pm$ 4.41 [720]
Child sex	Female	363/720	50.85%
	Male	357/720	49.15%
Time the child has been residing in the household (in months)			15.26 $\pm$ 5.34 [720]
Has the child ever attended EPI services?	Yes	711/720	98.47%
	No	9/720	1.53%
Has the child ever received IPTi-SP?^a,b^	Yes	638/707	88.96%
	No	69/707	11.04%
Vaccination coverage^b,c^ (EPI schedule in Sierra Leone)	Fully vaccinated	392/717	53.42%
	Partially vaccinated	314/717	44.69%
	Not vaccinated	11/717	1.88%
Vaccination coverage^b,d^ (WHO)	Fully vaccinated	474/717	64.03%
	Partially vaccinated	232/717	34.09%
	Not vaccinated	11/717	1.88%
Is the child taking cotrimoxazole?^b^	Yes	54/709	7.28%
	No	655/709	92.72%
Is the child taking antiretroviral therapy?^b^	Yes	18/707	2.48%
	No	689/707	97.52%
Did the child sleep inside a mosquito net the night before the interview?	Yes	558/720	77.31%
	No	162/720	22.69%
**Participants’ caretakers**		**n/N**	**Weighted Percentage or** **mean ± SD or median + IQR**
Caretaker’s age (in years)^b^			29.00(23.00–37.00) [710]
Caretaker’s sex^b^	Female	624/717	87.78%
	Male	93/717	12.22%
Caretaker’s relationship with the child^b^	Mother	556/717	78.11%
	Father	70/717	9.14%
	Other	91/717	12.75%
Caretaker’s main type of income^b^	No salary	79/717	11.21%
	Self-employment	611/717	85.17%
	Paid employment	27/717	3.63%
Caretakers with formal employment^b^	Formal employment No formal employment	27/717 690/717	3.63% 96.37%
Caretaker’s highest level of education^b^	Never attended school	395/718	54.69%
	Primary	78/718	10.75%
	Secondary or higher	245/718	34.56%
Is the caretaker able to read and write?	Illiterate	519/720	71.91%
	Partially literate	105/720	14.16%
	Fully literate	96/720	13.93%
Caretaker’s marital status^b^	Single (never married)	82/715	11.52%
	Married or in union	578/715	81.02%
	Separated or divorced	24/715	3.17%
	Widow/er	31/715	4.29%
Caretaker’s religion^f^	Christian	210/720	27.62%
	Muslim	508/720	72.10%
	None	2/720	0.28%
**Household head**		**n/N**	**Weighted Percentage**
Sex of the household head^b^	Female	392/719	55.00%
	Male	327/719	45.00%
Household head’s relationship with the child	Mother	234/720	32.78%
	Father	269/720	36.55%
	Other	217/720	30.67%
Household head’s main type of income^b^	No salary	51/719	7.30%
	Self-employment	614/719	85.36%
	Paid employment	54/719	7.34%

^a^Source of information: 74.69% child’s vaccination card or related document; 25.31% reported by caretaker

^b^Missing values: Variable has missing values

^c^Fully vaccinated child: defined as a child who received all vaccines scheduled from birth to 9 months in Sierra Leone’s national EPI schedule. Partially vaccinated child: defined as a child who received at least one, and not all, vaccines scheduled from birth to 9 months in Sierra Leone’s national EPI schedule. Child not vaccinated: defined as a child who has received no vaccine at the time of interview

^d^Fully vaccinated child: defined by the WHO as a child who received 1 dose of Bacillus de Calmette-Guérin (BCG) vaccine for tuberculosis, 3 doses of oral polio vaccine (OPV), 3 doses of pentavalent vaccines (Penta), and 1 dose of Measles-containing vaccine (MCV) before reaching one year old. Partially vaccinated child: defined as a child who received at least one of the following vaccines but not all before reaching one year old: 1 dose of BCG, 3 doses of OPV, 3 doses of Penta, and 1 dose of MCV. Child not vaccinated: defined as a child who has received no vaccine at the time of interview

^e^Formal employment: defined as full time or seasonal paid employment with formal salary. No formal employment: defined as no formal salary, includes self-employment and in-kind as type of incomes

^f^Traditional African religion 0.00%, Other religions 0.00%

Women were 87.78% of the caretakers (Table 1). Median age of the caretakers was 29 years, and in 78.11% of cases, the caretaker was the child’s mother. Among caretakers, 71.91% were unable to read and write, 96.37% of them did not have formal employment, while 81.02% of them were married or in union. Caretakers were identified as Muslims (72.10%), Christians (27.62%), or declared to have no religion at all (0.28%). Fifty per cent of the caretakers were household heads, and most household heads did not have formal employment (92.66%).

Most surveyed children attended the EPI services at least once (98.47%, 711/720). Information on IPTi and vaccine administration was either retrieved from the child’s vaccination card (74.69%,) or self-reported by the child’s caretaker (25.31%,).

A total of 53.42% children had received all vaccines scheduled from birth to 9 months of age (fully immunized) as per the national EPI schedule, while 64.03% of the surveyed children were fully immunized, as per WHO definition [16]. The majority of the children (77.31%) had slept under a mosquito net the night before the interview.

### IPTi coverage

**Fig. 3:**
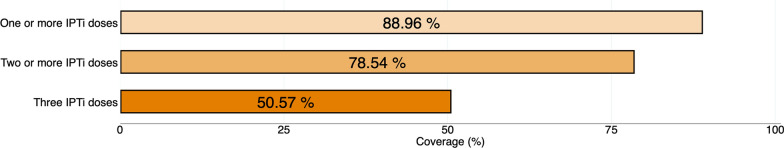
IPTi coverage in MULTIPLY project areas

Of the 720 children, 13 of them did not provide information regarding the IPTi intake. Among the remaining 707 children, 368 received three doses of IPTi (weighted proportion 50.57%; 95% CI 45.38–55.75), 197 received two doses of IPTi (weighted proportion 27.97%; 95% CI 23.84–32.50), and 73 received one dose only of IPTi (weighted proportion 10.42%; 95%CI 7.88–13.65), regardless of administration timepoints. Overall, 638 children received one or more IPTi doses (weighted proportion 88.96%; 95%CI 85.54–91.65), 565 received two or more doses (weighted proportion 78.54%; 95%CI 74.30–82.25) (Table 2, Fig. 3) and 69 (weighted proportion 11.04%; 95%CI 8.35–14.46) did not receive any IPTi dose (Table 2). By district, the coverage of three IPTi doses was 65.25% (95% CI 56.71–72.91) in Bombali, 50.60% (95% CI 38.78–62.35) in Tonkolili, and 38.52% (95% CI 30.80–46.87) in Port Loko.

**Table 2:** | IPTi coverage in study areas

	Only one dose	Only two doses	Three doses
A. Doses received, irrespective of EPI timepoint*			
Received (n/N)	73/707	197/707	368/707
Weighted Proportion (95% CI)	**10.42%** (7.88–13.65)	**27.97%** (23.84–32.50)	**50.57%** (45.38–55.75)

	One or more doses	Two or more doses	Three doses
B. Cumulative doses received, irrespective of EPI timepoint			
Received (n/N)	638/707	565/707	368/707
Weighted Proportion (95% CI)	**88.96%** (85.54–91.65)	**78.54%** (74.30–82.25)	**50.57%** (45.38–55.75)

	IPTi1 (10 weeks)	IPTi2 (14 weeks)	IPTi3 (9 months)
C. Doses received at each EPI contact			
Received (n/N)	574/707	577/707	420/707
Weighted Proportion (95% CI)	**80.26% **(75.30–84.44)	**80.09% **(76.30–83.40)	**57.72% **(53.20–62.11)

Source of information for Sections A and B: 75.68% child’s vaccination card or related document; 24.32% reported by caretaker; for Section C: IPTi1 (10 w) 74.58% child’s vaccination card or related document, 25.42% reported by caretaker; IPTi2 (14 w) 76.26% child’s vaccination card or related document, 23.74% reported by caretaker; IPTi3 (9 m) 75.86% child’s vaccination card or related document, 24.14% reported by caretaker

Penta2 (N = 6), OPV2 (N = 6), Pneumo3 (N = 6), Rota2 (N = 6), Penta3 (N = 6), OPV3 (N = 6), Pneumo3 (N = 6), IPV (N = 6), Yellow fever vaccine (N = 6), MCV1 (N = 6)

**Fig. 4:**
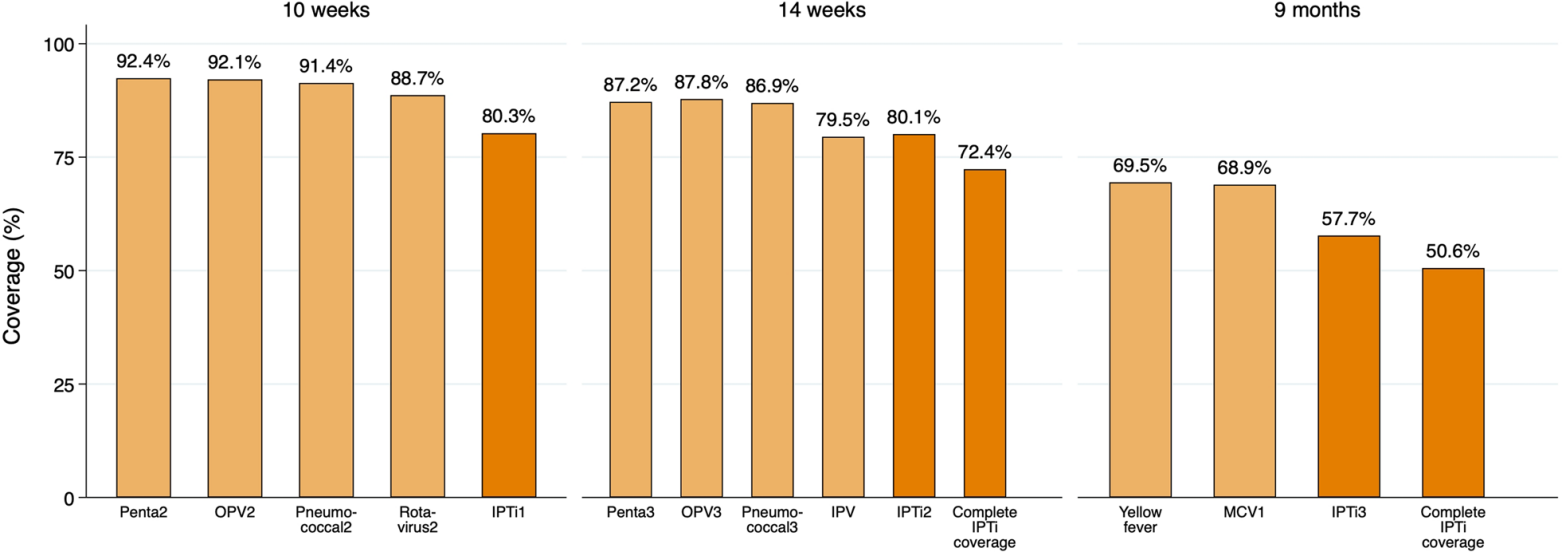
IPTi doses received at each EPI contact and vaccination coverage at the same timepoint.

According to the EPI schedule, 574 children received IPTi1 at 10 weeks of age (weighted proportion 80.26%; 95%CI 75.30–84.44), 577 children received IPTi2 at 14 weeks of age (weighted proportion 80.09%; 95%CI 76.30–83.40), and 420 children received IPTi3 at 9 months of age (weighted proportion 57.72%; 95%CI 53.20–62.11) (Table 2). Except for inactivated polio vaccine at 14 weeks of age, the coverage of IPTi was always lower than that of the vaccines administered at the same EPI visit (Fig. 4).

### Malaria prevalence

Overall, malaria prevalence was 28.19% (95% CI 24.81–31.84). By district, the prevalence was 23.26% (95% CI 17.51–30.21), 29.17% (24.44–34.39), and 35.71% (95% CI 28.62–43.49) in Bombali, Port Loko and Tonkolili, respectively. Overall, the prevalence of clinical malaria (fever or history of fever plus a positive RDT) was 17.10% (95% CI 14.08–20.62) (Additional file 2: Annex 2).

### Factors associated with malaria infection 

A substantial amount of variation across clusters was found; the variance of the random effects for cluster is 0.8330 (95%CI 0.1735–3.9996) in the adjusted model. Adjusted for all other studied covariates, older children were more likely to have malaria infection (OR per month increase 1.07, 95% CI 1.03 – 1.10, *P*-value 0.0004) (Table 3). Children who had received three doses of IPTi were less likely to have malaria infection although borderline at the 0.05 level (OR 0.62, 95% CI 0.38–1.02, *P*-value 0.0588). Compared with Bombali district, children residing in Tonkolili district were more likely to test positive for malaria (OR 2.03, 95% CI 1.24–3.30, *P*-value 0.0053).

**Table 3:** Logistic regression of factors potentially associated with malaria infection*

Variable^1^		Univariable models		Multivariable model	
		Crude OR (95% CI)	p-value	Adjusted OR (95% CI)	p-value
Child’s age in months		1.06 (1.03–1.10)	0.0009	1.07 (1.03–1.10)	0.0004
Child’s sex	Female	1	0.5474	1	0.5904
	Male	0.89 (0.61–1.30)		0.90 (0.62–1.31)	
Use of mosquito net at night		0.73 (0.50–1.06)	0.0923	0.94 (0.63–1.41)	0.7745
Three IPTi3 doses received		0.52 (0.36–0.76)	0.0008	0.62 (0.38–1.02)	0.0588
Fully immunized, WHO definition		0.52 (0.34–0.79)	0.0024	0.82 (0.48–1.41)	0.4701
Caretaker’s age in years		1.00 (0.98–1.01)	0.5832	1.00 (0.98–1.01)	07122
Caretaker’s sex	Female	1	0.7702	1	0.8664
	Male	0.93 (0.58–1.49)		0.96 (0.59–1.56)	
Caretaker’s relationship with the child	Parent	1	0.6840	1	0.5965
	Other	0.90 (0.52–1.54)		1.15 (0.68–1.93)	
Is the caretaker able to read and write?	Illiterate	1	0.7243 0.0204	1	0.3026 0.0131
	Partially literate	0.93 (0.62–1.40)		0.78 (0.48–1.26)	
	Fully literate	0.51 (0.29–0.90)		0.43 (0.22–0.83)	
Caretaker’s main type of income	No salary	1	0.6236 0.4589	**1**	0.7624 0.0421
	Self-employment	0.88 (0.51–1.50)		1.10 (0.59–2.02)	
	Paid employment	1.38 (0.58–3.26)		3.02 (1.04–8.75)	
Caretaker’s marital status	Single (never married)	1	0.9919 0.8557 0.5289	1	0.5433 0.8628 0.3166
	Married or in union	1.00 (0.59–1.70)		0.82 (0.42–1.58)	
	Separated or divorced	1.11 (0.37–3.30)		0.90 (0.26–3.10)	
	Widow/er	0.70 (0.23–2.14)		0.53 (0.15–1.87)	
Caretaker’s religion	Christian	1	0.7974 0.6713	1	0.0846 0.8553
	Muslim	0.95 (0.65–1.39)		0.70 (0.46–1.05)	
	Other	1.86 (0.10–34.63)		1.27 (0.09–17.58)	
District	Bombali	1		**1**	
	Tonkolili	2.10 (1.32–3.33)	0.0021	2.03 (1.24–3.30)	0.0053
	Port Loko	1.52 (1.00–2.34)	0.0524	1.54 (0.97–2.43)	0.0675
Locality	Rural	1	0.8369	1	0.4122
	Urban	1.05 (0.67–1.62)		0.83 (0.54–1.29)	

CI confidence interval, *OR* odds ratio

^1^The first listed category of each variable was taken as reference value. In case of dummy variables, “No” was taken as a reference value

^*^n = 683 observations

### Factors associated with IPTi coverage

Similar to the adjusted model with malaria infection as outcome variable, a substantial amount of variation across clusters was found; the variance of the random effects for cluster is 0.8965 (95%CI 0.6140–1.3092). Adjusted for all other studied covariates, older children (OR per month increase 1.07, 95% CI 1.02–1.11, *P*-value 0.0056), those who slept under a mosquito net the previous night (OR 1.76, 95% CI 1.22–2.53, *P*-value 0.0029) and those whose caretaker was paid-employed (OR 2.74, 95%CI 1.11, 6.74, *P-*value 0.0290) were more likely to have received the complete three IPTi doses (Table 4). Children with a positive RDT result (OR 0.57, 95% CI 0.39–0.82, *P*-value 0.0035), children whose caretakers were males (OR 0.50, 95% CI 0.28–0.91, *P*-value 0.0251) and of Muslim religion (OR 0.51, 95% CI 0.32–0.81, *P*-value 0.0045) and children residing in Port Loko district (OR 0.40, 95% CI 0.19–0.87, *P*-value 0.0212) were less likely to have received complete three doses of IPTi.

**Table 4:** Logistic regression of factors potentially associated with three IPTi doses*

Variable^1^		Univariable models		Multivariable model	
		Crude OR (95% CI)	p-value	Adjusted OR (95% CI)	p-value
Child’s age in months		1.07 (1.02–1.12)	0.0044	1.07 (1.02–1.11)	0.0056
Child’s sex	Female	1	0.6286	1	0.2450
	Male	0.93 (0.70–1.24)		0.83 (0.61–1.14)	
Positive RDT-result		0.52 (0.35–0.77)	0.0013	0.57 (0.39–0.82)	0.0035
Use of mosquito net at night		2.01 (1.40–2.89)	0.0003	1.76 (1.22–2.53)	0.0029
Caretaker’s age in years		1.01 (1.00–1.03)	0.0184	1.00 (0.98–1.01)	0.8381
Caretaker’s sex	Female	1	0.3450	1	0.0251
	Male	0.77 (0.45–1.33)		0.50 (0.28–0.91)	
Caretaker’s relationship with the child	Parent	1	0.0273	1	0.6897
	Other	1.68 (1.06–2.67)		1.12 (0.63–2.02)	
Is the caretaker able to read and write?	Illiterate	1	0.5293 0.4161	1	0.8501 0.7876
	Partially literate	0.86 (0.54–1.37)		0.95 (0.56–1.62)	
	Fully literate	1.26 (0.72–2.23)		1.08 (0.61–1.89)	
Caretaker’s main type of income	No salary	**1**	0.2262 0.0869	**1**	0.2258 0.0290
	Self-employment	0.71 (0.40–1.25)		0.70 (0.39–1.26)	
	Paid employment	2.30 (0.88–6.00)		2.74 (1.11–6.74)	
Caretaker’s marital status	Single (never married)	1	0.1739 0.0617 0.0038	1	0.5115 0.2918 0.1470
	Married or in union	1.43 (0.85–2.41)		1.21 (0.68–2.16)	
	Separated or divorced	2.79 (0.95–8.17)		1.86 (0.58–5.98)	
	Widow/er	4.07 (1.60–10.33)		2.02 (0.78–5.25)	
Caretaker’s religion	Christian	1	0.3841 0.1258	1	0.0045 0.9579
	Muslim	0.82 (0.53–1.29)		0.51 (0.32–0.81)	
	Other	0.18 (0.02–1.64)		1.11 (0.02–55.32)	
District	Bombali	**1**		**1**	
	Tonkolili	0.57 (0.23–1.44)	0.2309	0.68 (0.26–1.78)	0.4271
	Port Loko	0.33 (0.16–0.66)	0.0023	0.40 (0.19–0.87)	0.0212
Locality	Rural	1	0.3616 0.4678	1	0.7922
	Urban	0.77 (0.37–1.58)		1.11 (0.50–2.45)	

CI confidence interval, OR odds ratio

^1^The first listed category of each variable was taken as reference value. In case of dummy variables, “No” was taken as a reference value

^*^n = 698 observations

## Discussion

This study assessed the coverage of IPTi and malaria prevalence in children U2 in selected districts of Sierra Leone before the initiation of a pilot implementation project to expand IPTi administration into the 2 year of life, as it has recently been recommended by WHO [8]. The findings show that half of children aged 10 to 23 months had received the three recommended doses of IPTi (50.57%, 95% CI 45.38–55.75), while malaria infection and clinical (symptomatic) malaria were present in less than a third (28.19%) and less than a quarter (17.10%) of them, respectively.

Both complete IPTi and IPTi 3 coverages in this survey (50.57% and 57.72%, respectively) are lower than that (59%) found in the same districts and nation-wide in the latest malaria indicator survey (MIS) undertaken nation-wide by the Ministry of Health and Sanitation during the peak of the malaria season (July–September 2021) (unpublished observation, NMCP). Both figures are also higher than the 32.2% coverage of complete IPTi reported in 2018. Regarding malaria prevalence it was 43.65% by RDT in these districts, reflecting the higher transmission intensity during the MIS 2021 compared to that when this survey was carried-out at the beginning of the dry season.

Alike the observation on coverage of vaccines and IPTi doses at each EPI contact in this study, in the 2018 survey the proportion of children who received IPTi at each timepoint was also lower than vaccination rates at the same EPI contact. These findings indicate that although IPTi has been successfully integrated into the EPI program in Sierra Leone, some limitations still exist that represent missed opportunities for some children attending the EPI scheme who do not receive the recommended malaria prevention at the same visit. Similarly, regarding malaria control in pregnancy it has been also reported missed opportunities in the proportion of pregnant women receiving intermittent preventive treatment in pregnancy (IPTp), being systematically lower than the proportion of women attending the antenatal clinic visit [17]. As with IPTp, some reasons may explain the observed misalignment between IPTi uptake and the immunizations administered, namely, SP stock-outs, children -in the case of IPTi-, presenting with acute diseases (such as clinical malaria), lack of equipment or water to administer the drug, or caretaker´s refusal, or lack of staff commitment. All of this suggest that the lack of adherence to the national EPI schedule is not the only barrier to achieve optimal IPTi coverage rates. Further work is needed to identify challenges in the delivery of malaria preventive interventions in children and pregnant women.

In this survey, infants who had received three doses of IPTi were less likely to have malaria infection, indicating the effectiveness of the intervention in preventing malaria. On the other hand, older age was associated with a higher risk of the child having malaria infection at the time of the interview. This may be explained by the fact that the last IPTi dose is usually administered at around 9 months of age. Since the post-prophylactic effect of SP is about 5 weeks, older infants and children may not be protected against malaria compared to younger children. For this reason, the WHO has released new guidelines recommending the expansion of IPTi or PMC into the second year of life [8]. The EPI contact point to administer the booster vaccination against measles (usually between 15 and 18 months of age) will facilitate the expansion of IPTi. Alike in the last MIS national survey (2021), children from Tonkolili district were more likely to have malaria infection than children from the other two districts, reflecting the higher malaria transmission in Tonkolili (Unpublished observation, NMCP Sierra Leone).

Older age was associated with increased probability of having received the three IPTi doses. This may be due to the fact that older children have had more time to attend the EPI program and, albeit delayed, received all intervention doses. Children who slept under a mosquito net the night before were also more likely to have received the three IPTi doses, probably reflecting a positive health behaviour. On the other hand, children in Port Loko district were less likely to have received the complete course of IPTi. This finding coincides with the results from the 2019 Sierra Leone’s Demographic and Health Survey, showing Port Loko district to have the lowest immunization coverage in the country [18]. The WHO-UNICEF estimations of measles 2 coverage for SL is 50–67% for 2016–2021. In this survey, however, the observed coverage of Measles 2 vaccination among the 456 15–23 month year old children surveyed is 40.2% (95%CI: 34.6–46.1). Modelling analysis to look at the potential impact of expanded doses of PMC alongside the different EPI contacts when it is administered will be useful to assess the potential impact of the intervention on malaria prevalence at each contact.

This study has some limitations. First, the list of selected clusters was based on the 2015 census with two of the clusters being outside project areas, for which they had to be replaced by backup clusters, leading to potential underrepresentation of part of the area, and thus to potential non-response bias. Secondly, although information on child’s IPTi and immunization status was obtained from the child’s vaccination card in most cases, there is a potential for recall bias when this information was reported by the caretaker.

The strengths of this study are the use of a robust multi-stage cluster sampling approach to select a representative sample of children U2 from areas where the MULTIPLY project will be implemented. Field teams underwent a rigorous training, a 2 day pre-test, and a feedback session before the start of the HHS. In addition, data collection was carefully supervised by the investigators team. This maximized compliance with the sampling methodology, standard operating procedures, and reduced the potential for selection bias and protocol deviations. The use of electronic devices for direct data entry in the field ensured data quality by eliminating errors in data transfer from paper forms while quality rules were automatically executed in mobile devices. Lastly, the rate of non-response among eligible children was 1.1%, much lower than the 10% estimated during sample size calculation, thus minimizing the potential for non-response bias at individual level.

## Conclusion

The results of this study show an increase in IPTi coverage since the pilot evaluation carried out in 2018. Although these findings are encouraging, just half of the surveyed children had received the recommended full IPTi course. In addition, uptake misalignment between IPTi and concomitant vaccines given at the same EPI contacts persist. Improving young children’s health by reducing their malaria burden requires increasing efforts to promote and expand malaria prevention coverage into the second year of life.

## Supplementary Information

**Additional file 1.** MULTIPLY community-based household survey standard operating procedure.

**Additional file 2.** Malaria prevalence.

## Data Availability

The dataset is deposited at CORA.RDR (Catalan Open Research Area. Repositori de dades de Recerca), available through the following link: https://doi.org/10.34810/data603.
