# Supplementary material for: Coverage of intermittent preventive treatment of malaria in infants after four years of implementation in Sierra Leone
Source: Malar J. 2023 May 2;22:145. doi: 10.1186/s12936-023-04575-6 (PMC10151216; doi:10.1186/s12936-023-04575-6)
Supplement: Supplementary file 1 — Additional file 1. MULTIPLY community-based household survey standard operating procedure. [file 12936_2023_4575_MOESM1_ESM.docx]

**ANNEX 1**

**MULTIPLY community-based household survey
Standard Operating Procedure**

| **SOP code** | SOP_HHS_001_v01_EN |
| --- | --- |
| **Version** | v01 |
| **Language** | EN |
| **Title** | Multi-stage sampling methodology |
| **Written by & Date** | Cesc Bertran-Cobo, 13 September 2021 |
| **Revised by & Date** | Mireia Llach, 27 September 2021 |
| **Approved by & Date** |  |
| **Approval signature** |  |
| **Original version language** |  |
| **Translated by & Date** |  |

| **Date** | **Amendment** | **Approved by & signature** | **Reason for the amendment** |
| --- | --- | --- | --- |
|  |  |  |  |
|  |  |  |  |
|  |  |  |  |

1. **objective**

The objective of this SOP is to describe the three-stage cluster sampling method that will be used to select the administrative units from MULTIPLY project areas that will be included in the community-based HHS.

1. **acronyms and abbreviations**

| **EPI** | Expanded Programme on Immunization |
| --- | --- |
| **HF** | Health Facility |
| **HHS** | Household survey |
| **ICF** | Informed Consent Form |
| **PPS** | Probability Proportional to Size |
| **SOP** | Standard Operating Procedure |
| **U5** | Under five years of age |

1. **definitions**

| **Cluster** | Any sampling unit (temporal or geographical in nature) in which one or more administrative units (villages, towns, cities…) can be associated. |
| --- | --- |
| **Cluster sample size** | Number of children to be interviewed in each cluster. It will be **12 children** in all clusters. |
| **Household** | Group of people who live together, sharing the same cooking pot. |
| **Sampling frame** | List of all cities, towns, villages, and any other administrative units located within the project area, with as up-to-date population data as possible. |
| **Sampling interval** | Interval used to systematically select clusters from the sampling frame. |
| **Survey sample size** | Number of children to be enrolled in the HHS (Sierra Leone = 710; Togo = 682; Mozambique = 770). |

1. **applicable to**

All MULTIPLY project staff responsible for three-stage cluster sampling method to select the administrative units from MULTIPLY project areas that will be included in the community-based HHS.

1. **responsibilities**

**HHS coordinator**: Must ensure that the procedures provided here are followed as described.

**All study personnel**: Must be familiar with the content of this SOP and follow it for all participant’s procedures in the context of the HHS.

1. **supplies, materials and equipment**

- Photocopy of the project area’s sampling frame
- Photocopy of the Cluster Control Sheet
- Photocopy of the Household Control Sheet
- Photocopy of the Survey Profile
- Photocopy of a table of random numbers
- Project tablets
- Pen
- Paper
- Folder

1. **SAMPLING METHODOLOGY**

A household cluster survey method will be followed to randomly select candidate children from MULTIPLY project areas to be invited to participate in the HHS. The sampling will be done in 3 stages:

1. Identification and random selection of survey clusters;
2. Random selection of households; and
3. Random selection of the child to be approached in each randomly selected household.
   1. **First stage: Identifying and selecting survey clusters**

The aim of this first stage is to randomly select the clusters to be surveyed in the project area, applying a Probability Proportional to Size (PPS) sampling approach to ensure that the most populated clusters have an increased probability of being selected. The steps to follow are:

- - 1. **Obtaining the sampling frame**

**Sampling frame**: list of all smallest administrative units available located within the project area, with as up-to-date population data as possible.

Obtain the sampling frame for your MULTIPLY project area with the collaboration of community leaders and local authorities^^[[1]](#footnote-1)^^.

- - 1. **Listing all the villages**

Using the sampling frame, create a list of all smallest administrative units within your project area, indicating:

- Distance to the nearest HF where MULTIPLY is implemented / hard to reach area,
- Each unit’s population, and
- The cumulative population.

The list must be sorted by distance / classification. Administrative units that are known not to be accessible for any reason before the implementation of the study (insecurity, flooding, etc) will not be included in the list.

- - 1. **Calculating the N of clusters**

The number of clusters in each project country is calculated by using the following equation:

$$N clusters=\frac{survey sample size}{cluster sample size}$$

The number of clusters to be randomly selected is indicated in Table 1:

**Table 1: N of clusters to be randomly selected, per country**

| **Country** | **Survey sample size** | **Cluster sample size** | **N** **of clusters** |
| --- | --- | --- | --- |
| Sierra Leone | 710 | 12 | 60 |
| Togo | 682 | 12 | 57 |
| Mozambique | 770 | 12 | 65 |

- - 1. **Calculating the sampling interval**

**Sampling interval**: interval used to systematically select clusters from the sampling frame, calculated by dividing the total population of the sampling frame by the number of clusters calculated in step 7.1.3, rounding off the result to the nearest whole number.

Calculate the sampling interval by using the following equation:

$$Sampling interval=\frac{total population of the sampling frame}{N clusters}$$

In this equation, the **total population of the sampling frame** is divided by the **N of clusters** calculated in Step 7.1.3.

- - 1. **Applying PPS**

Apply the PPS sampling approach, to give the most populated clusters an increased probability of being selected (Figure 1):

1. With use of Excel or any other available software, randomly generate a number **between 1** and the **sampling interval**.
2. Find the first administrative unit in the list created in Step 7.1.2, in which the cumulative population equals or exceeds the random number. This will be the first cluster you will select.
3. Select the following cluster by **adding** the **sampling interval** to the **randomly generated number**.
4. Repeat the previous procedure as many times as necessary until you have selected the number of clusters calculated in step 7.1.3.

**Figure 1: Practical example for the random selection of clusters**


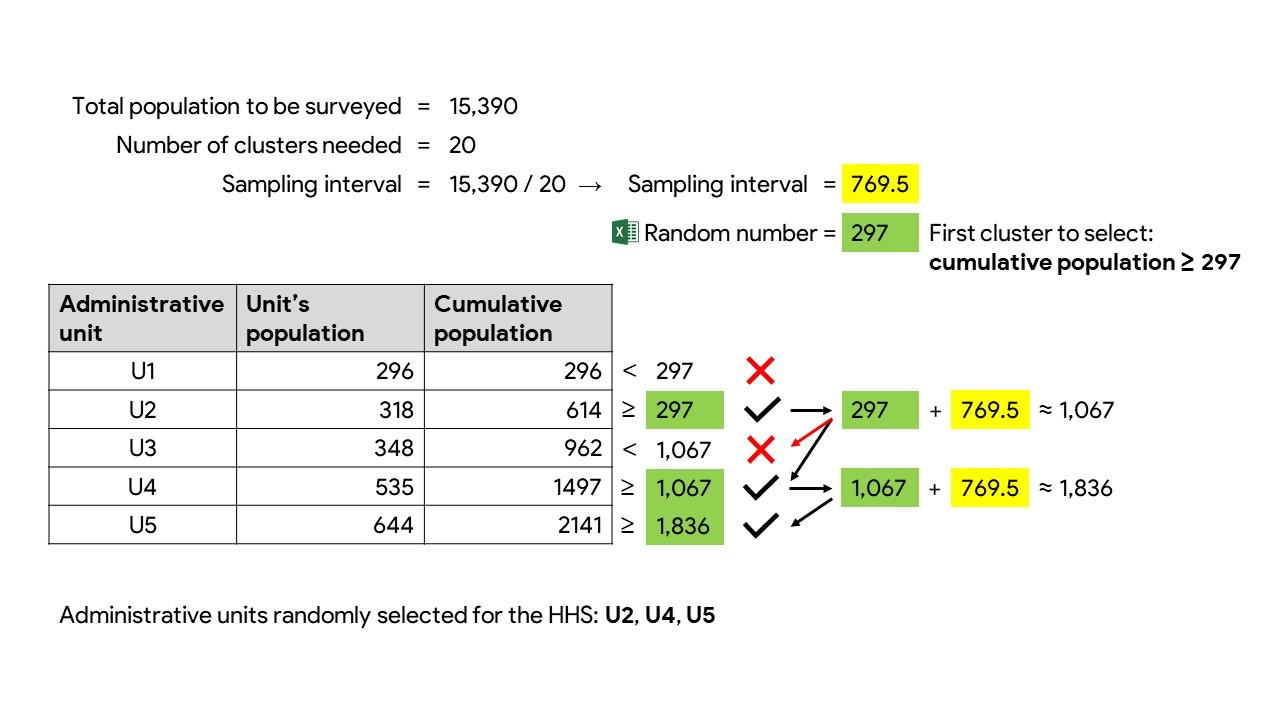


In large administrative units, it can happen that after doing the PPS, more than one cluster is allocated in the same unit. See second stage sampling to know how to proceed in this situation.

- - 1. **Backup clusters**

Some backup clusters have to be selected to be used in case some of the clusters selected in the previous steps are not accessible during data collection (due to flooding, rainy season, insecurity, or any previously unforeseen reasons). Backup clusters must be selected from the remaining **units from the sampling frame not selected** in steps 7.1.4 and 7.1.5.

Remove from the sampling frame the clusters selected in previous steps before doing the selection of the backup clusters. The number of backup clusters needed will be estimated by the survey coordinators from each country but it could be considered around 10% of the number of clusters calculated in step 7.1.3.

Once the back-up clusters are selected, randomly select the order in which they must be surveyed.

- 1. **Second stage: Selection of households**

The aim of this second stage is to randomly select the households to be surveyed in each selected cluster.

**Household**: group of people who live and eat together, sharing the same cooking pot.

The steps to follow in this second stage are:

- - 1. **Listing the households**

Generate a census of the households located in each selected cluster. You may face one of the following situations during this step, actions to be taken are described accordingly:

1. A complete census / list of all households is available in the randomly selected cluster.

Action:

1. Assign a number to each household, with use of the *Cluster control sheet (SOP_HHS_001_A02).*
2. There is no census and the cluster size is about 100 households or less.

Action:

- 1. Revise and/or draw a map of the households of the cluster with the help of an informant;
  2. Make a list or map with the location of each household (see pictures in Annex 9.2); and
  3. Assign a number to each household, with use of the *Cluster control sheet (SOP_HHS_001_A02)*.

1. There is no census and the cluster size is above 100 households.

Action:

- 1. Revise and/or draw a general map of the cluster with the help of an informant;
  2. Subdivide the cluster into two or more similar sections of around 100 households each (depending on how large is the cluster);
  3. Give a number to each section;
  4. Randomly select a section using the *Table of random numbers, with instructions (SOP_HHS_001_A04)*; and
  5. Assign a number to each household, with use of the *Cluster control sheet (SOP_HHS_001_A02)*.

For those cases where a house or building is composed by more than one household, **give a** **number to all of them** before doing the random selection (step 7.2.2).

NOTE: If there is **more than one cluster in one administrative unit**, the instructions below must be followed before selecting the household:

1. Divide unit in segments of similar size, of about 100 households each, using existing elements such as rivers or main roads (whenever possible) (see pictures in Annex 9.1),
2. Give a number to each segment,
3. Randomly select the total number of allocated clusters from these segments, and
4. Continue with the household selection of each selected segment according to the different situations described above, steps 7.2.1 b) to d).

**Figure 2 | Practical examples of an administrative unit segmentation**


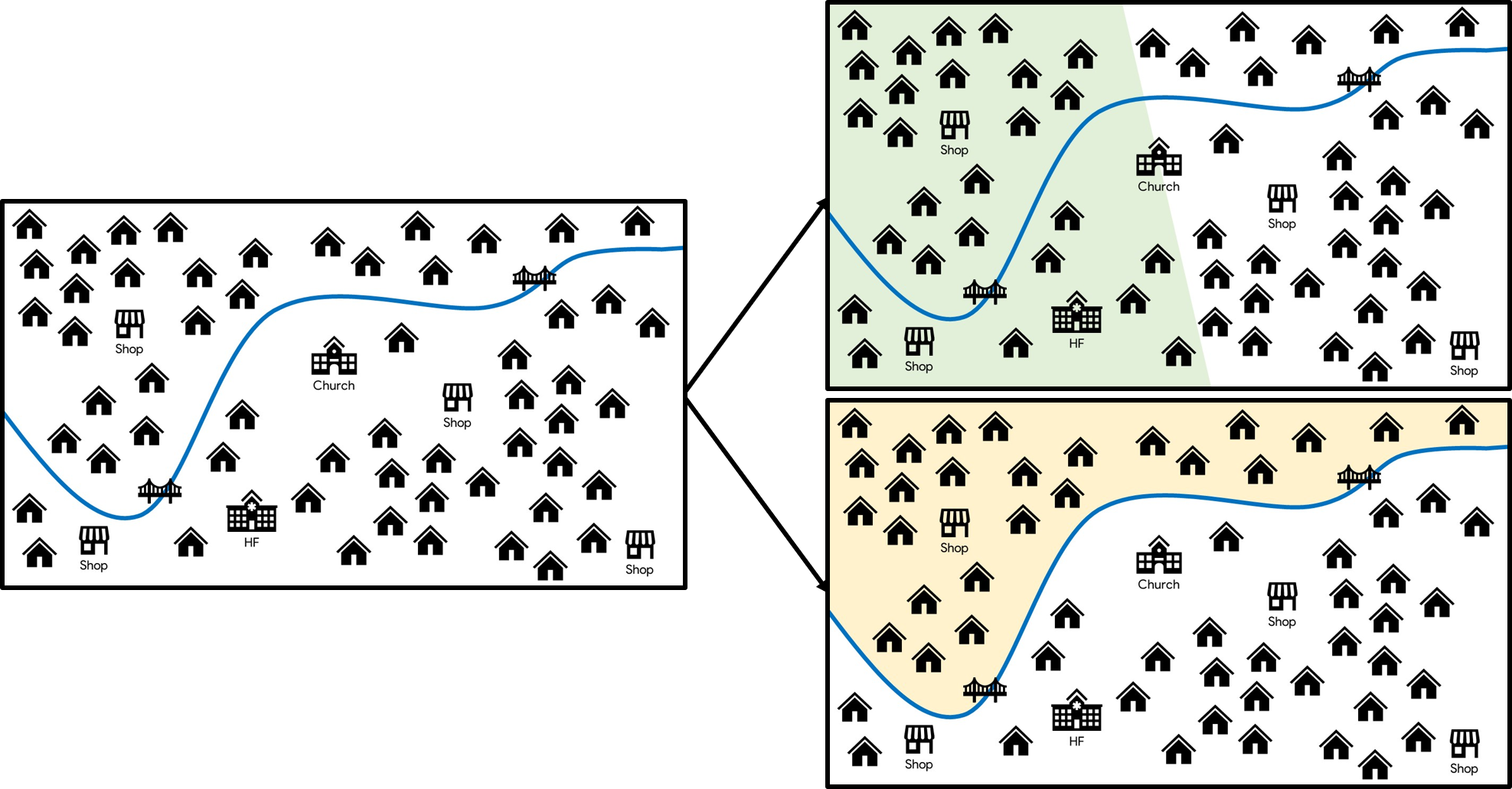


- - 1. **Selecting the households**

Once all identified households have been assigned a number (ID), the households to be visited will be selected randomly. The number of households to be visited in order to find the eligible children will be defined by the survey coordinators in each country as it can be context-specific. To randomly select the households, several methods can be valid but it is important to ALWAYS use the same method in ALL clusters.

If all households in a cluster are visited and the recruitment goal (i.e., cluster sample size) has not been met, the nearest administrative unit will be visited. The closest households to the previous cluster will be visited until the number is achieved.

- 1. **Third stage: Selection of children to be recruited**

The aim of this third stage is to randomly select the child to be approached for recruitment in the selected household, amongst all eligible children. The steps to follow in this third stage are:

- - 1. **Listing all children**

The first person that you should approach is the household head in the selected household. Explain him/her the survey objectives and ask for his/her oral permission to perform the random selection of the child. The household head will also be interviewed to obtain a list of all children U5 living in the household. List all children in the *Household control sheet (SOP_HHS_001_A03)*.

If the household head is not available, any other adult that agrees to provide the information can be also interviewed if the socio-cultural context allows.

- - 1. **Assigning a number to all eligible children**

Give a number to all **eligible** children living in the household (i.e., all children who **meet** all of the **inclusion criteria**).

- - 1. **Selecting the child**

Randomly select one eligible child among all eligible ones with use of the *Table of random numbers, with instructions (SOP_HHS_001_A04)*. If there is only one child in the household that meets the inclusion criteria, select that child.

- - 1. **Approaching the child’s caretaker**

Once a child has been selected, the caretaker must be approached for inform consent process and interview.

You may face one of these situations during this step, please act accordingly:

1. The caretaker is available OR is absent but is nearby, within 30 minutes’ walk.

Action:

- 1. Go find the him/her with the help of a guide from the community;
  2. If him/her is found, proceed with introduction of the study. Before proceeding with the inform consent form (ICF) and the interview make sure the selected child will be also available as we will have to perform the malaria RDT (see bullet points c and d).

1. The caretaker is absent and far away, more than 30 minutes’ walk.

Action:

- 1. DO NOT select another eligible child from the same household;
  2. Go to the next selected household or schedule a revisit. Possibility and number of revisits will be considered and agreed by the survey coordinators in each country and the same approach must be followed in all clusters.

1. The caretaker doesn't want to sign the ICF or is not able to respond due to any health/physical condition.

Action:

- 1. DO NOT interview him/her, and DO NOT select another eligible child from the same household;
  2. Go to the next selected household.

1. The caretaker would like to sign the ICF but the child is not around.

Action:

- 1. If possible, schedule a revisit later when the child can be found. Possibility and number of revisits will be considered and agreed by the survey coordinators in each country and the same approach must be followed in all clusters
  2. If not possible to schedule a re-visit, DO NOT interview them, and DO NOT select another eligible child from the same household;
  3. Go to the next selected household.

1. It is revealed during the interview that the selected child does not really meet the inclusion criteria (i.e., he/she is not eligible).

Action:

- 1. Randomly select another eligible child from the same household, if there are any;
  2. Follow the instructions for any of the situations described in 7.3.4 a) to d), if applies.

1. **after multi-stage sampling has been performed**

Once a child has been selected and we have ensured that s/he and the caretaker are available the caretaker must be approached for inform consent process and interview. Please read and follow the *Informed consent, inclusion criteria screening, and enrolment in MULTIPLY HHS* (*SOP_HHS_002)* to conduct this part of the HHS procedures.

1. **ANNEXES AND REFERENCE DOCUMENTS**

SOP_HHS_001_A01 Sampling frame

SOP_HHS_001_A02 Cluster control sheet

SOP_HHS_001_A03 Household control sheet

SOP_HHS_001_A04 Table of random numbers, with instructions

- 1. **Example picture of cluster segmentation**


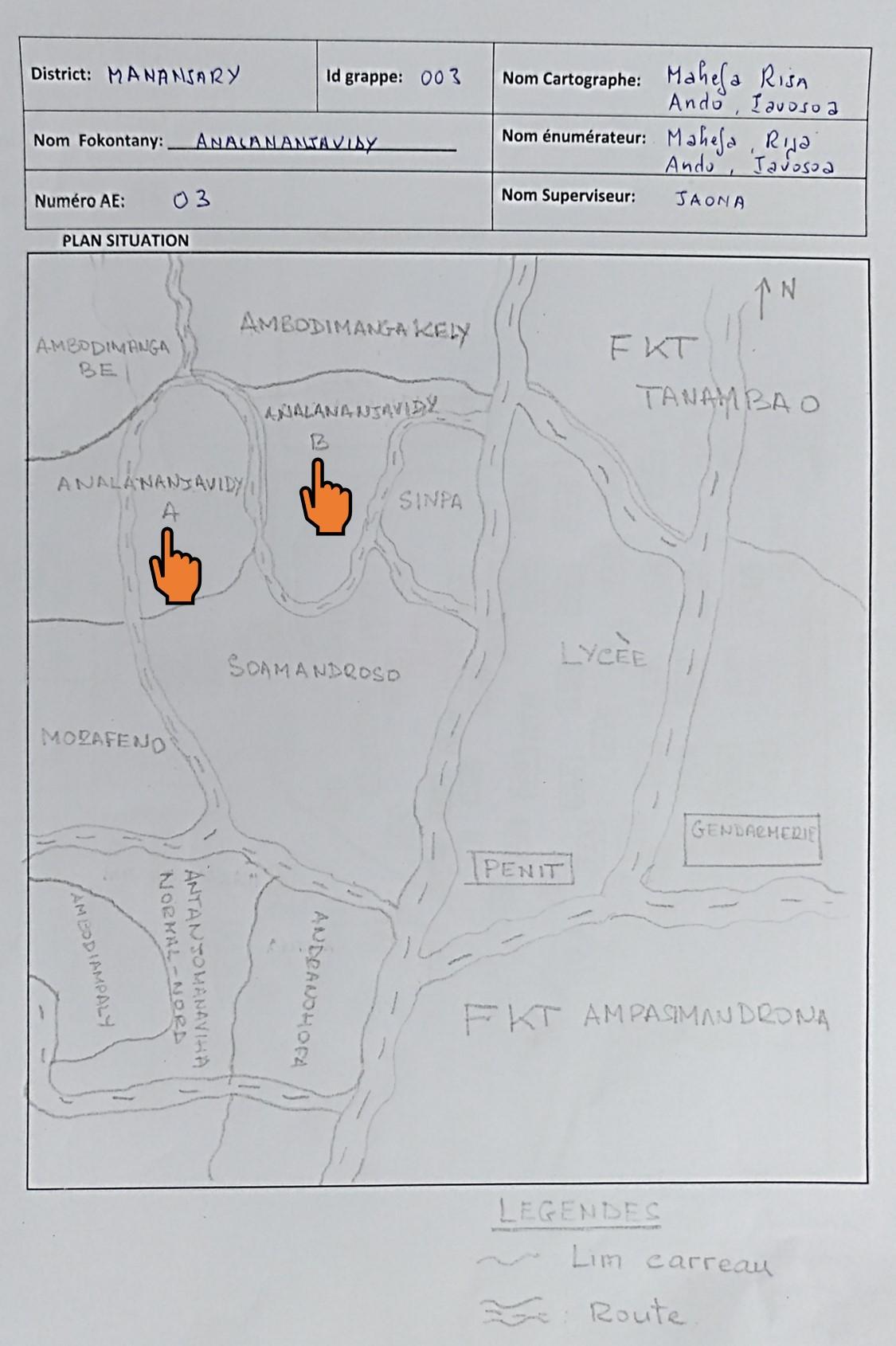


- 1. **Example pictures of household mapping**


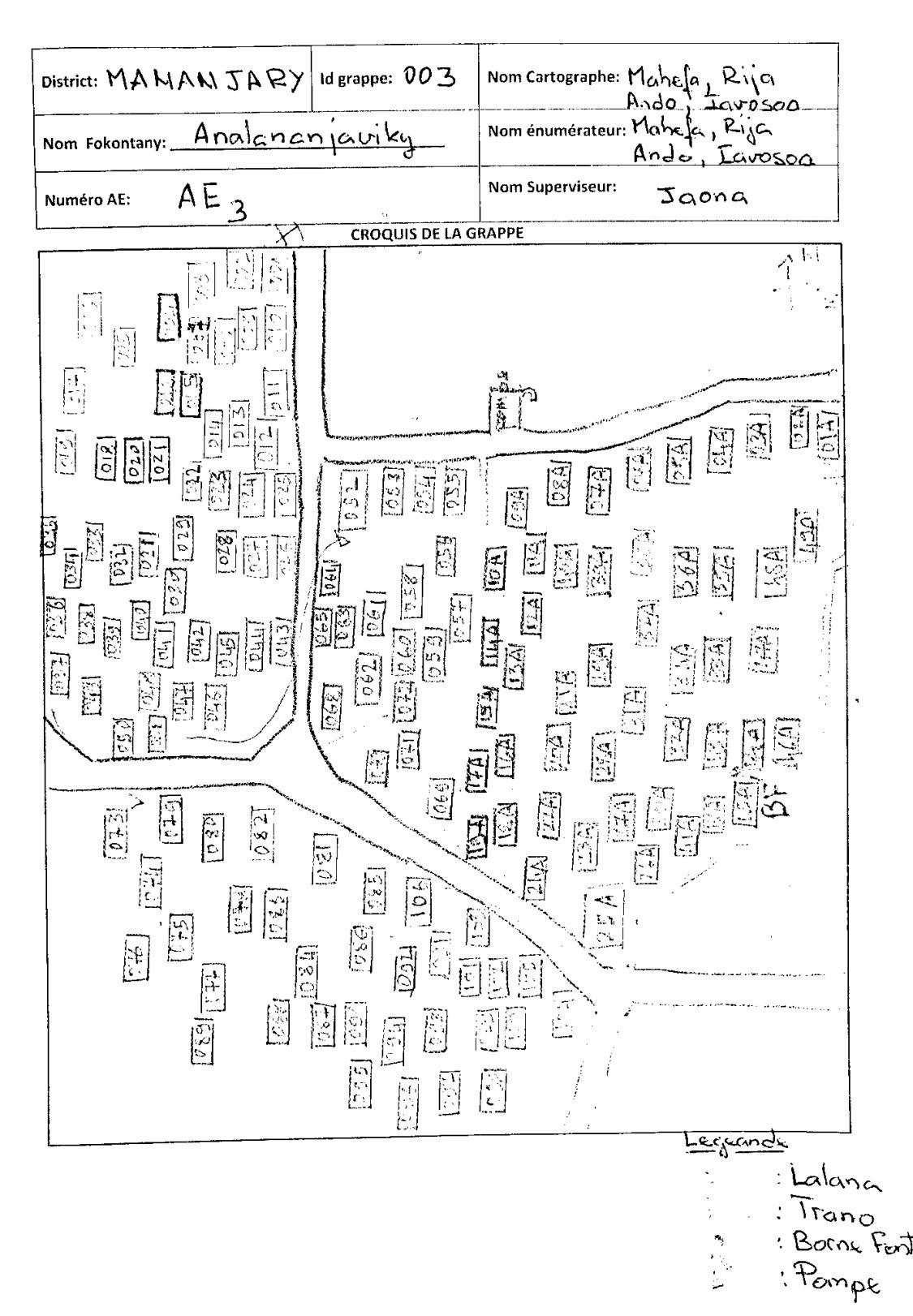


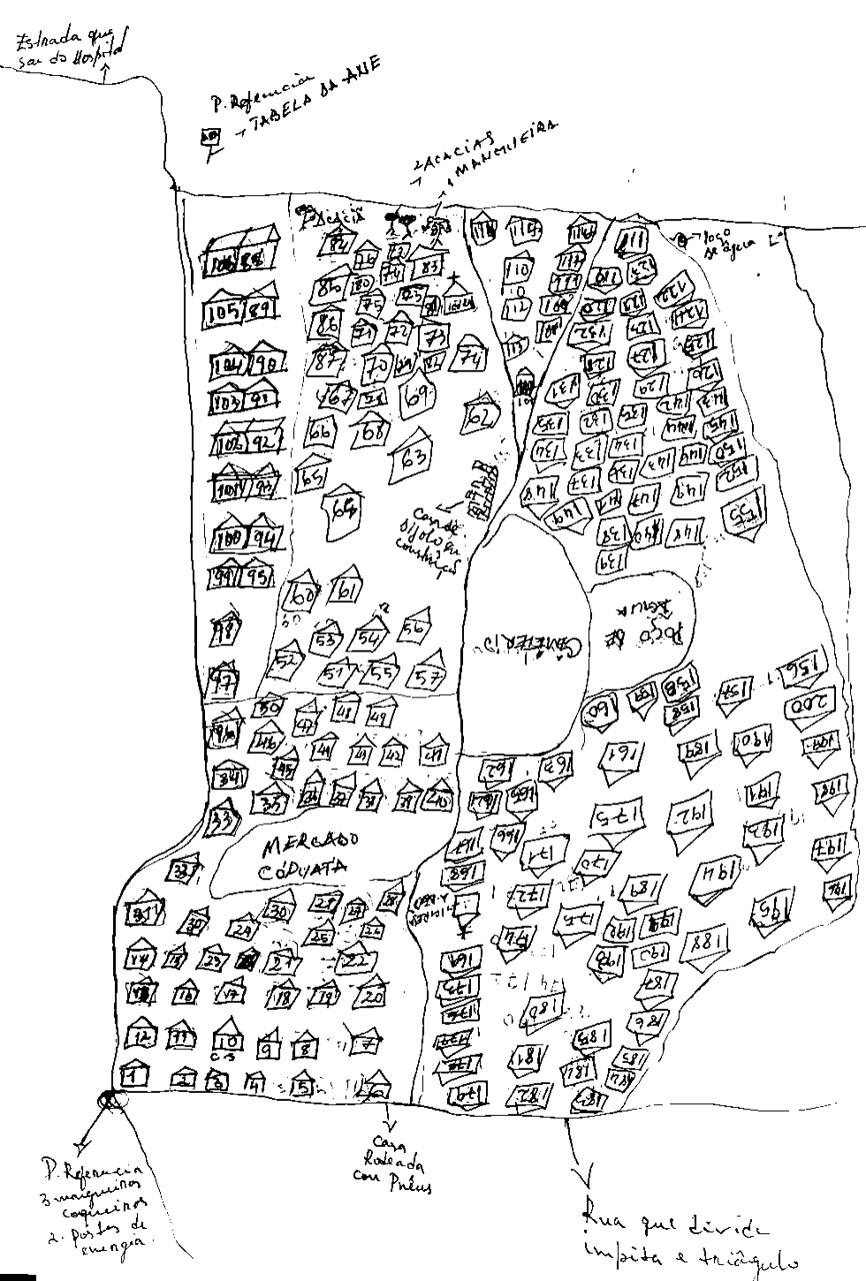


1. Specifically for Sierra Leone: Catchment areas belonging to ICARIA recruitment sites will be excluded from MULTIPLY’s sampling frame and multi-stage sampling methodology. [↑](#footnote-ref-1)
