# Supplementary material for: Coverage of intermittent preventive treatment of malaria in infants after four years of implementation in Sierra Leone
Source: Malar J. 2023 May 2;22:145. doi: 10.1186/s12936-023-04575-6 (PMC10151216; doi:10.1186/s12936-023-04575-6)
Supplement: Supplementary file 2 — Additional file 2. Malaria prevalence. [file 12936_2023_4575_MOESM2_ESM.docx]

**ANNEX 2**

**MALARIA PREVALENCE**

| **Variable** | | **District** | | | **Total (N=720)** |
| --- | --- | --- | --- | --- | --- |
|  |  | **Bombali (N=288)** | **Port Loko (N=264)** | **Tonkolili (N=168)** |  |
| RDT ^1^ | Negative | 221 [76.74%; (69.79, 82.49)] | 187 [70.83%; (65.61, 75.56)] | 108 [64.29%; (56.51, 71.38)] | 516 [71.81%; (68.16, 75.19)] |
|  | Positive | 67 [23.26%; (17.51, 30.21)] | 77 [29.17%; (24.44, 34.39)] | 60 [35.71%; (28.62, 43.49)] | 204 [28.19%; (24.81, 31.84)] |
|  | Indeterminate | 0 (0.00%) | 0 (0.00%) | 0 (0.00%) | 0 (0.00%) |
|  | *Total* | *288 (100.00%)* | *264 (100.00%)* | *168 (100.00%)* | *720 (100.00%)* |
| Clinical malaria ^1^ | | 34 / 288 [11.81%; (7.68, 17.72)] | 51 / 263 [19.39%; (14.69, 25.15)] | 37 / 167 [22.16%; (15.62, 30.44)] | 122 / 718 [17.10%; (14.08, 20.62)] |
